# Second haploidentical stem cell transplantation (HAPLO-SCT2) after relapse from a first HAPLO-SCT in acute leukaemia—a study on behalf of the Acute Leukaemia Working Party (ALWP) of the European Society for Blood and Marrow Transplantation (EBMT)

**DOI:** 10.1038/s41409-023-01985-7

**Published:** 2023-05-09

**Authors:** Giuliano Filippini Velázquez, Myriam Labopin, Johanna Tischer, Anna Maria Raiola, Emanuele Angelucci, Alexander D. Kulagin, Piero Galieni, Arancha Bermúdez, Claude-Eric Bulabois, Nicolaus Kröger, José Luis Díez-Martín, Mi Kwon, Arnon Nagler, Christoph Schmid, Fabio Ciceri, Mohamad Mohty

**Affiliations:** 1 Section for Stem Cell Transplantation, Augsburg University Hospital and Medical Faculty, Augsburg, Germany; 2 EBMT Paris Study Unit, Saint-Antoine Hospital, Paris, France; 3 Department of Haematology, Hôpital Saint-Antoine, Sorbonne University, INSERM UMRs 938, Paris, France; 4 Department of Internal Medicine III, University Hospital of Munich, Campus Grosshadern, Munich, Germany; 5 Hematology and cellular therapy unit, IRCCS Ospedale Policlinico San Martino, Genova, Italy; 6 RM Gorbacheva Research Institute, Pavlov University, St. Petersburg, Russian Federation; 7 Haematology Service, Mazzoni Hospital, Ascoli Piceno, Italy; 8 Servicio de Hematología-Hemoterapia, Hospital Universitario Marqués de Valdecilla, Santander, Spain; 9 Service d’Hématologie, CHU Grenoble Alpes—Université Grenoble Alpes, Grenoble, France; 10 University Medical Center Hamburg-Eppendorf, Department of Stem Cell Transplantation, Hamburg, Germany; 11 Sección de Trasplante de Médula Ósea, Hospital Gregorio Marañón, Madrid, Spain; 12 Hematology and Bone Marrow Transplant Unit, Chaim Sheba Medical Center, Tel Hashomer, Israel; 13 Unit of Hematology and Bone Marrow Transplantation, IRCCS San Raffaele Scientific Institute, Vita-Salute San Raffaele University, Milan, Italy

**Keywords:** Leukaemia, Stem-cell therapies

## Abstract

For patients with acute myeloid and lymphoblastic leukaemia (AML/ALL) lacking a matched sibling or unrelated donor, haploidentical stem cell transplantation (HAPLO-SCT) is increasingly used. However, available data on the treatment of relapse after HAPLO-SCT, including feasibility and efficacy of a second HAPLO-SCT (HAPLO-SCT2), is scarce. Hence, adults with AML/ALL, that had undergone HAPLO-SCT2 without ex-vivo manipulation after haematologic relapse from HAPLO-SCT1 were selected for a retrospective registry analysis. Eighty-two patients (AML, *n* = 63, ALL, *n* = 19, median follow-up: 33 months) were identified. Engraftment rate was 87%. At day +180, cumulative incidences of acute GvHD II-IV°/chronic GvHD were 23.9%/22.6%, respectively. Two-year overall survival/leukaemia-free survival (OS/LFS) were 34.3%/25.4%; 2-year non-relapse mortality (NRM) and relapse incidence (RI) were 17.6% and 57%. Leukaemia was the most frequent cause of death. Separated by disease, 2-year OS/LFS/NRM/RI were 28.7%/22.3%/16.2%/61.6% in AML, and 55.3%/38.4%/23.5%/38.2% in ALL patients. In a risk-factor analysis among patients with AML, stage at HAPLO-SCT1 and HAPLO-SCT2, and interval from HAPLO-SCT1 to relapse significantly influenced outcome. Our data demonstrate that HAPLO-SCT2 is a viable option in acute leukaemia relapse after HAPLO-SCT1. Engraftment, toxicity, risk factors and long-term outcome are comparable to data reported after allo-SCT2 in a matched donor setting.

## Introduction

Acute myeloid and acute lymphoblastic leukaemia (AML/ALL) represent the most common indications for allogeneic haematopoietic stem cell transplantation (allo-SCT) in Europe [1]. The availability of HAPLO-SCT has expanded transplant options for patients lacking a human leucocyte antigen (HLA)-matched sibling or unrelated donor (MSD/MUD) [2–5] and is increasingly used [6]. Comparative analyses have shown that haploidentical (HAPLO) donor for first allo-SCT (allo-SCT1) in different stages of AML and ALL is a suitable alternative to MUD or MSD, associated with comparable results [7–23].

For patients with acute leukaemia (AL) that relapse after allo-SCT1, a second allogeneic transplantation (allo-SCT2) represents a viable treatment option, especially in patients without relevant co-morbidities, who relapse >6 months from the first transplant. Historically, allo-SCT2 using either MSD or MUD after an allo-SCT1 from MSD/MUD could achieve long term survival independently of donor type for allo-SCT2 and donor change. Among others [24–26], this was reported by Christopeit et al. [27] who found 2-year overall survival (OS) and leukaemia-free survival (LFS) rates of 25% and 21%, after allo-SCT2 from MSD and MUD in a multicenter analysis including 179 patients. In the study of Kharfan-Dabaja et al., [28] 2- and 5-year OS after allo-SCT2 in 137 AML patients was 26%/19%, respectively. More recently, Nagler et al. [29] analysed the outcome of 245 ALL patients limited to MSD/MUD for allo-SCT2 and observed 2-year/5-year LFS rates of 20%/12%, and 2-year/5-year OS of 30%/14%.

With the increasing routine use of HAPLO-SCT, HLA-mismatched family donors were also used more frequently for second transplants [30]. Tischer et al. [31] reported on 20 consecutive patients with AL achieving 1-year OS/LFS of 45%/33% and non-relapse mortality (NRM) of 36% after sequential conditioning HAPLO-SCT2 for relapse following matched donor SCT1. Shimoni et al. [32] investigated the outcome of 556 patients after allo-SCT2 for AML relapse following matched SCT1 by dividing patients into three groups: same donor [*n* = 163, MSD/MSD-112, MUD/MUD-51], different matched donor [*n* = 305, MSD/different MSD-44, MSD/MUD-93, MUD/different MUD-168], or HAPLO [*n* = 88, MSD/HAPLO-45, MUD/HAPLO-43]. Two-year OS/LFS rates were 36.4%/23.5%, 28.7%/23.7%, and 23.3%21.8%, respectively, with no statistically significant differences among cohorts. However, on multivariate analysis, HAPLO-SCT2 was associated with higher NRM. In two further studies performed on behalf of the Acute Leukaemia Working Party (ALWP) of the European Society for Blood and Marrow Transplantation (EBMT), Kharfan-Dabaja et al. reported comparable outcomes after allo-SCT2 from either MUD or HAPLO, both in AML [33] and ALL [34].

Thus, available data suggest comparable outcomes after allo-SCT2, regardless of donor type. However, in the vast majority, these analyses were based on patients that had received their first transplant from either a MSD or a MUD. Patients that had received two HAPLO-SCT represented a small minority at best, without a separate analysis. Hence, data supporting the feasibility and efficacy of HAPLO-SCT2 in patients who had developed leukaemia relapse after being transplanted from a HAPLO donor at allo-SCT1 is scarce [35, 36]. In this registry-based study we report on the outcome of 82 patients with AL that underwent HAPLO-SCT2 after relapse from HAPLO-SCT1 across EBMT centres.

## Methods

### Study population

This was a registry-based analysis of adults transplanted for AML and ALL. Eligible patients had to have received in-vivo T-cell replete HAPLO-SCT2 after haematologic relapse from a T-cell replete HAPLO-SCT1 between 2007 and 2021. Patients receiving HAPLO-SCT2 for other reasons such as graft failure were excluded, as were patients receiving ex-vivo T cell depleted grafts at either first or second HAPLO-SCT. Data were provided by the ALWP registry of the EBMT, which is a voluntary working society that collects data from more than 600 transplant centres. All participants are required to report all consecutive hematopoietic SCT including follow-up once a year. Regular audits are performed to check for data accuracy.

The protocol was approved by the ALWP general assembly and conducted in accordance with the Declaration of Helsinki and Good Clinical Practice guidelines. All patients had provided written informed consent authorizing the use of their data for research purposes.

### Statistics

Outcomes of interest were engraftment, OS, LFS, NRM, cumulative relapse incidence (RI), graft-versus-host disease (GvHD), and GvHD-free, relapse-free survival (GRFS). All outcomes were measured from the time of HAPLO-SCT2. Patient-, disease- and treatment-related characteristics at the time of HAPLO-SCT1, relapse and HAPLO-SCT2 were summarized using median and range for continuous, and frequency and percentage for categorical data. The Kaplan-Meier method was used to estimate OS, LFS and GRFS. Cumulative incidence functions were used for RI and NRM in a competing risk setting, while death and relapse were included as competing events when calculating the cumulative incidence of GvHD. All surviving patients were censored at the time of last documented contact. Univariate analyses were done using Gray’s test for cumulative incidence functions and the log-rank test for survival analyses. For univariate analyses, continuous variables were categorized and the median value was used as a cut-off point. The number of patients was not sufficient to allow for a reliable multivariate analysis which was therefore not performed. All p-values were two-sided, and values <0.05 were considered statistically significant. Statistical analyses were performed with SPSS 25.0 (SPSS Inc, Chicago, IL, USA) and R 4.0.2 (R Core Team 2020).

### Definitions

Complete remission (CR) was defined by bone marrow (BM) blasts <5%, absence of circulating blasts, absence of extramedullary disease, and haematologic recovery [37]. Relapse refers to BM blasts ≥5%, reappearance of blasts in peripheral blood or development of extramedullary disease [37]. Engraftment was defined as the first of three consecutive days with an absolute neutrophil count of ≥500 cells/μL. OS was defined as time from HAPLO-SCT2 to death. LFS was defined as time from HAPLO-SCT2 to either death or relapse/progression after HAPLO-SCT2. Following HAPLO-SCT2, NRM was defined as death without evidence of relapse/progression, GRFS was defined as alive status with neither grade III-IV acute GvHD, no-systemic therapy-requiring chronic GvHD, nor relapse or death [38]. The intensity of the conditioning regimen was classified based on established criteria [39]. Risk scoring of AML was performed according to the 2017 recommendations by the European Leukaemia Network (ELN) [37].

## Results

### Patients’ characteristics

A total of 82 patients (AML, *n* = 63; ALL, *n* = 19, median year of HAPLO-SCT2: 2018) were identified. The median age at HAPLO-SCT2 was 47.2 [range (r): 18.3–69.3] years for AML and 33.5 (r: 19.7–58.2) years for ALL. The median interval from HAPLO-SCT1 to relapse was 7.5 months (r: 0.8–59.4). A change of donor between HAPLO-SCT1 and HAPLO-SCT2 was chosen in 35 patients (63% of informative cases) with AML and 17 (90%) of patients with ALL. At start of conditioning for HAPLO-SCT2, 42 (67%) of AML patients and 7 (37%) of ALL patients had active disease. Myeloablative/reduced intensity conditioning (MAC/RIC) was used for HAPLO-SCT2 in 34 (43%) and 45 (57%) of patients, and post-transplant cyclophosphamide (PTCy) was the most common basis for GvHD prophylaxis (*n* = 54, 82%). See Table 1 for detailed patient-, disease- and treatment-related characteristics of HAPLO-SCT1 and 2.

**Table 1:** Patients characteristics.

	Overall (*n* = 82)	AML (*n* = 63)	ALL (*n* = 19)
*HAPLO-SCT1*			
Patient age (years) at HAPLO-SCT1			
median (range)	42.1 (18.2–66.9)	44.8 (18.2–66.9)	31.4 (19–57.5)
Patient sex			
male	46 (56.1%)	36 (57.1%)	10 (52.6%)
female	36 (43.9%)	27 (42.9%)	9 (47.4%)
Donor sex			
donor male	52 (63.4%)	39 (61.9%)	13 (68.4%)
donor female	30 (36.6%)	24 (38.1%)	6 (31.6%)
Year of HAPLO-SCT1	2016 (2002–2021)	2016 (2002–2021)	2016 (2010–2019)
Stage at HAPLO-SCT1			
CR1	37 (45.7%)	26 (41.9%)	11 (57.9%)
CR2	14 (17.3%)	9 (14.5%)	5 (26.3%)
CR3	1 (1.2%)	1 (1.6%)	0 (0%)
advanced	29 (35.8%)	26 (41.9%)	3 (15.8%)
missing	1	1	0
ELN risk classification (AML)*			
low risk		3 (5%)	
intermediate risk		29 (46%)	
adverse risk		18 (29%)	
NA/failed		13 (20%)	
Subtype ALL			
Ph negative B-lineage ALL			7 (36%)
Ph positive B-lineage ALL			2 (11%)
T-linieage ALL			8 (42%)
missing			2 (11%)
Stem cell source at HAPLO-SCT1			
BM	28 (34.1%)	19 (30.1%)	9 (47.4%)
PB	52 (63.4%)	42 (66.7%)	10 (52.6%)
BM + PB	2 (2.4%)	2 (3.2%)	0 (0%)
Female to male combination			
no female to male	66 (80.5%)	49 (77.8%)	17 (89.5%)
female to male	16 (19.5%)	14 (22.2%)	2 (10.5%)
Patient CMV			
patient CMV negative	16 (20.8%)	13 (21.7%)	3 (17.6%)
patient CMV positive	61 (79.2%)	47 (78.3%)	14 (82.4%)
missing	5	3	2
Donor CMV			
donor CMV negative	26 (33.3%)	23 (37.7%)	3 (17.6%)
donor CMV positive	52 (66.7%)	38 (62.3%)	14 (82.4%)
missing	4	2	2
Conditioning intensity before HAPLO-SCT1			
MAC	44 (53.7%)	32 (50.8%)	12 (63.2%)
RIC	38 (46.3%)	31 (49.2%)	7 (36.8%)
GVHD prevention for HAPLO-SCT1			
PTCy	57 (73.1%)	43 (71.7%)	14 (77.8%)
in-vivo TCD	17 (21.8%)	15 (25%)	2 (11.1%)
both	4 (5.1%)	2 (3.3%)	2 (11.1%)
missing	4	3	1
*HAPLO-SCT2*			
Follow-up after HAPLO-SCT2 (months)			
median (95% CI)	33	38.03 (24.98–56.63)	18.87 (7.17–48.12)
Patient age (years) at HAPLO-SCT2			
median (range)	43.4 (18.3–69.3)	47.2 (18.3–69.3)	33.5 (19.7–58.2)
Year of HAPLO-SCT2			
median (range)	2018 (2007–2021)	2018 (2007–2021)	2018 (2011–2021)
Time from HAPLO-SCT1 to relapse (months)	7.5 (0.8–59.4)	8.7 (0.8–59.4)	6.7 (1-56.4)
median (range)			
Time from HAPLO-SCT1 to HAPLO-SCT2 (months)			
median (range)	16.3 (1.0–91.5)	15.2 (1.0–91.5)	18.4 (1.5–61.6)
Stage at HAPLO-SCT2			
CR	33 (40.2%)	21 (33.3%)	12 (63.2%)
active disease	49 (59.8%)	42 (66.7%)	7 (36.8%)
Stem cell source at HAPLO-SCT2			
BM	15 (18.3%)	10 (15.9%)	5 (26.3%)
PB	67 (81.7%)	53 (84.1%)	14 (73.7%)
Donor change from HAPLO-SCT1 to HAPLO-SCT2			
yes	52 (62.6%)	35 (62.5%)	17 (89.5%)
no	23 (27.7%)	21 (37.5%)	2 (10.5%)
missing	7	7	0
Donor sex			
donor male	48 (58.5%)	39 (61.9%)	9 (47.4%)
donor female	34 (41.5%)	24 (38.1%)	10 (52.6%)
Female to male donor combination			
no female to male	63 (76.8%)	50 (79.4%)	13 (68.4%)
female to male	19 (23.2%)	13 (20.6%)	6 (31.6%)
Patient CMV			
paient CMV negative	16 (19.8%)	14 (22.6%)	2 (10.5%)
patient CMV positive	65 (80.2%)	48 (77.4%)	17 (89.5%)
missing	1	1	0
Donor CMV			
donor CMV negative	30 (37%)	26 (41.9%)	4 (21.1%)
donor CMV positive	51 (63%)	36 (58.1%)	15 (78.9%)
missing	1	1	0
Karnofsky performance score at HAPLO-SCT2			
<90%	38 (51.4%)	29 (50%)	9 (56.2%)
≥90%	36 (48.6%)	29 (50%)	7 (43.8%)
missing	8	5	3
HCT-CI at HAPLO-SCT2			
HCT-CI = 0	36 (61%)	27 (60%)	9 (64.3%)
HCT-CI = 1 or 2	10 (16.9%)	7 (15.6%)	3 (21.4%)
HCT-CI ≥ 3	13 (22%)	11 (24.4%)	2 (14.3%)
missing	23	18	5
Conditioning intensity before HAPLO-SCT2			
MAC	34 (43%)	24 (40%)	10 (52.6%)
RIC	45 (57%)	36 (60%)	9 (47.4%)
missing	3	3	0
GvHD prevention for HAPLO-SCT2***			
PTCy	54 (81.8%)	40 (78.4%)	14 (93.3%)
in-vivo TCD	10 (15.2%)	9 (17.6%)	1 (6.7%)
both	2 (3%)	2 (3.9%)	0 (0%)
missing	16	12	4

*AML* acute myeloid leukaemia, *ALL* acute lymphoblastic leukaemia, *HAPLO-SCT1* first haploidentical stem cell transplantation, *HAPLO-SCT2* second haploidentical stem cell transplantation, *Ph* Philadelphia, *BM* bone marrow, *PB* peripheral blood, *MAC* myeloablative conditioning, *RIC* reduced intensity conditioning, *HT-CI* Hematopoetic cell transplantation—comorbidity index, *CR* complete remission, *CMV* cytomegalovirus, *PTCy* post-transplant cyclophosphamide, *TCD* T-cell depletion.

*2017 recommendations of the European Leukaemia Network (ELN).

***In addition to a calcineurin inhibitor ± methotrexate (MTX) or mycophenolate mofetil (MMF).

### Engraftment and GvHD rates

Overall, 87% of patients engrafted. Median time to engraftment was 18 days; engraftment rate by day +60 was 86% (CI 95%: 75.2 - 92). Cumulative incidences of acute GvHD grades II-IV and III-IV by day +180 were 23.9% (CI 95%: 14.7–34.4) and 15.3% (CI 95%: 8.1–24.6), respectively. The cumulative incidences of chronic GvHD and extensive chronic GvHD at 2 years were 22.6% (CI 95%: 13.6–32.9) and 11.2% (CI 95%: 5.2–19.8), respectively.

### Outcome

**Fig. 1:**
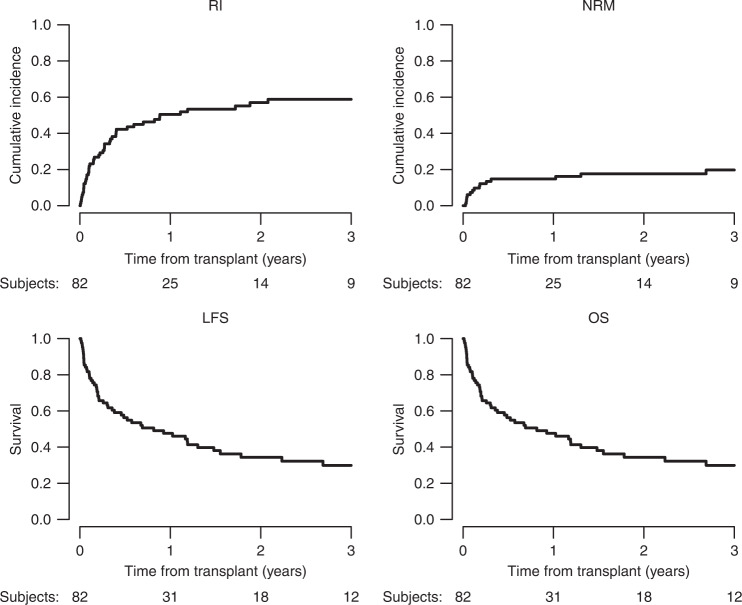
Outcome after second HAPLO-SCT in 82 patients with acute leukaemia. RI relapse incidence, NRM non-relapse mortality, LFS leukaemia-free survival, OS overall survival.

Median follow-up after HAPLO-SCT2, was 38 months (95% CI: 24.9–56.3) for AML and 19 months (95% CI: 7.1–48.1) for ALL patients. Kaplan-Meier estimates showed a 2-year OS and LFS for the entire cohort of 34.3% (CI 95%: 23.3–45.6) and 25.4% (CI 95%: 16–35.9), respectively. Two-year NRM and RI rates were 17.6% (CI 95%: 10.1–26.8) and 57% (CI 95%: 44.7–7.5) respectively (Fig. 1). The 2-year GRFS was 15.1% (CI 95%: 8–24.4).

When analysed separately by diagnosis, 2-year OS/LFS were 28.7% (CI 95%: 17.5–41)/ 22.3% (CI 95%: 12.5–33.8) for AML and 55.3% (CI 95%: 26.3–76.9)/ 38.4% (CI 95%: 16.2–60.4) for ALL, respectively. Two-year NRM for AML was 16.2% (CI 95%: 8.2–6.5) and 23.5% (CI 95%: 6.4–46.7) for ALL patients, while 2-year RI was 61.6% (CI 95%: 47.4–73) for AML and 38.2% (CI 95%: 16.1–60.2) for ALL patients. Consequently, leukaemia was the most frequent cause of death in both disease subgroups (*n* = 33, 62.3%). Other causes of death included infection in 12 (22.6%) and GvHD in 3 (5.7%) patients, whereas one patient each died from haemorrhage, graft failure, veno-occlusive disease, and post-transplant lymphoproliferative disease.

### Univariate analyses of risk factors

An analysis of risk factors for OS, LFS, NRM and RI at 2 years was performed among the 63 patients with AML. Variables of significance for OS were stage at HAPLO-SCT1 (active disease vs CR; *p* = 0.008), stage at HAPLO-SCT2 (*p* = 0.047) and interval from HAPLO-SCT1 to relapse [≤ vs > median (8.74 months); *p* = 0.001]. The same variables were significant for LFS (see Table 2 for details). Myeloablative conditioning for HAPLO-SCT2 was of borderline significance for improved LFS compared to RIC (p = 0.053), without increasing NRM. Variables that reached significance for RI were stage at HAPLO-SCT2 (*p* = 0.003) and interval from HAPLO-SCT1 to relapse (*p* = 0.008), while MAC showed a trend towards lower RI as compared to RIC (*p* = 0.08). For NRM, advanced stage at HAPLO-SCT1 (*p* = 0.09) and donor change for HAPLO-SCT2 showed a trend towards higher NRM (*p* = 0.052). Beyond, the effect of donor change for HAPLO-SCT2 was investigated both within the entire group of patients with AML, and among patients with late relapse, i.e., beyond 6 months from HAPLO-SCT1. The latter cut off was chosen due to the increased frequency of HLA loss as basic mechanism of relapse reported among patients with late relapse, leading to most extensive loss of a graft-versus-leukaemia effect if the same donor is used for HAPLO-SCT2 [30, 36, 40]. However, there was no significant influence of donor change on outcome, neither among all AML patients, nor among those with late relapse (donor change, *n* = 37 vs same donor, *n* = 12). Detailed information of univariate analyses is shown in Table 2. We did not perform univariate analyses for ALL due to the limited number of patients in this cohort. Similarly, numbers of AML patients were considered insufficient for a reliable multivariate analysis.

**Table 2:** Univariate analyses of risk factors for 2-year OS, LFS, NRM, and RI (AML, *n* = 63).

	*n*	OS (CI 95%)	LFS (CI 95%)	NRM (CI 95%)	RI (CI 95%)
Adverse cytogenetics^a^					
not adverse	45	31.9% (18.4–46.3)	24.6% (12.9–38.4)	15.7% (6.8–27.9)	59.7% (42.9–73.1)
adverse	18	21.7% (5.7–44.1)	16.3% (3.2–38.5)	17.2% (3.7–39.1)	66.5% (34.4–85.5)
*P* value		0.56	0.62	0.6	0.68
Secondary AML					
de novo	52	25.3% (13.7–38.7)	20% (10–32.4)	19.7% (10–31.8)	60.3% (44.7–72.8)
secondary AML	11	45.5% (16.7–70.7)	30.3% (5.9–60.4)	0%	69.7% (21.5–91.9)
*P* value		0.2	0.3	0.1	0.57
Stage at HAPLO-SCT1					
active disease	26	7.7% (1.3–21.7)	7.7% (1.3–21.7)	15.4% (4.6–32)	76.9% (53.6–89.5)
CR	36	44.7% (26.8–61.1)	31.4% (16.2–47.8)	17.4% (6.9–32)	51.2% (32.2–67.3)
*P* value		0.008	0.026	0.65	0.059
Stage at HAPLO-SCT2					
active disease	42	18.5% (8–32.2)	15.9% (6.7–28.6)	12.2% (4.3–24.5)	72% (54.9–83.5)
CR	21	52.2% (27.8–71.8)	35.5% (14.4–57.5)	24.6% (8.5–45)	39.9% (16.3–62.8)
*P* value		0.047	0.043	0.09	0.003
Patient age (years)					
≤44.8 (median)	32	24.3% (10.9–40.7)	19.5% (7.7–35.3)	12.5% (3.8–26.6)	68% (47.2–82)
>44.8	31	32.9% (16–50.9)	24.8% (10.5–42.1)	20% (7.8–36.2)	55.2% (34.1–72)
*P* value		0.2	0.27	0.67	0.23
Karnofsky performance score					
<90	29	27.6% (12.2–45.5)	25.3% (10.9–42.6)	21.5% (8.3–38.6)	53.3% (32.5–70.2)
≥90	29	35% (18–52.5)	23% (9.7–39.7)	13.8% (4.2–29)	63.2% (41.8–78.6)
*P* value		0.3	0.22	0.63	0.57
Conditioning for HAPLO-SCT2					
MAC	24	35.2% (16.7–54.3)	30.8% (13.5–50)	17% (5–35)	52.2% (29.3–71)
RIC	36	20% (7.7–36.5)	17.1% (6.7–31.5)	16.8% (6.7–30.9)	66.1% (46.9–79.7)
*P* value		0.3	0.053	0.78	0.08
Interval from HAPLO-SCT1 to relapse (months)					
≤8.74 (median)	32	9.9% (2.5–23.3)	9.4% (2.4–22.3)	15.6% (5.4–30.8)	75% (55–87.1)
>8.74	31	49.3% (29–66.7)	35.5% (17.8–53.8)	16.4% (5.8–31.8)	48% (27.1–66.3)
*P* value		0.001	0.001	0.71	0.008
Donor change for HAPLO-SCT2					
Yes	35	27% (12.7–43.5)	21.7% (9.4–37.2)	23.5% (10.7–39)	54.9% (35.7–70.5)
No	21	35.7% (15.5–56.6)	24.5% (7.7–46.1)	4.8% (0.3–20.4)	70.7% (40.3–87.6)
*P* value		0.76	0.5	0.052	0.35
Donor change for HAPLO-SCT2 in relapses ≥ 6 months after HAPLO-SCT1					
Yes	37	36.3% (22.1–50.6)	26.6% (14.9–39.9)	22% (11.6–34.6)	51.3% (36–64.7)
No	12	35.1% (15.5–55.6)	24.2% (7.7–45.6)	8.7% (1.4–24.7)	67.1% (38–84.8)
*P* value		0.56	0.83	0.16	0.39

*OS* overall survival, *LFS* leukaemia-free survival, *NRM* non-relapse mortality, *RI* relapse incidence, *MAC* myeloablative conditioning, *RIC* reduced intensity conditioning, *CR* complete remission.

^a^Unknown was considered as not adverse.

## Discussion

To our knowledge, this is the largest systematic analysis of patients with acute leukaemias undergoing HAPLO-SCT2 after relapse from a HAPLO-SCT1 reported so far. Nevertheless, over a period of 14 years, only 82 patients were identified within one of the largest available transplant registries. With the median year of HAPLO-SCT2 being 2018, this might be a consequence of less frequent use of HAPLO-SCT across Europe during the first years of our study period. The increasing use of HAPLO-SCT2 over time mirrors the gain of centres’ experience and optimization of supportive therapy in the setting of HAPLO-SCT. These aspects were reflected in the analyses from Shouval et al. [41] who recently reported a continuously improved outcome of HAPLO-SCT over the last two decades. However, the relatively low number of identified patients in our study might also reflect a general reluctance of transplanting physicians to expose their patients to a second HAPLO-SCT, most likely due to the expectation of increased toxicity or graft failure rates. Anyhow, according to our data, toxicity, GvHD rates as well as engraftment after HAPLO-SCT2 were comparable to results published after second transplants both from HLA matched donors and from HAPLO donors after matched SCT-1. In particular, the cumulative incidence of NRM was surprisingly low (17% at two years), underscoring the feasibility of a HAPLO-SCT2 after HAPLO-SCT1.

When analysed separately, outcome seemed to be better among patients suffering from ALL (2y-OS 55%, 2y-LFS 38%) as compared to AML. These results should however be interpreted with caution, as in our cohort, ALL patients were younger, more often received HAPLO-SCT2 in CR and from a new donor, and median follow-up was only 19 months. Nevertheless, the data are in concordance with the improvement over time of adult ALL patients after PTCy-based HAPLO-SCT1 [42] and HAPLO-SCT2 following matched-allo-SCT1 [34]. The integration of targeted therapies such as tyrosine kinase inhibitors, bispecific antibodies, or antibody-drug conjugates into both first line and salvage treatment of ALL might also have contributed to these recent improvements.

An analysis of possible risk factors for outcome was performed within the larger cohort of patients with AML. In univariate analyses, stage at HAPLO-SCT1 and HAPLO-SCT2, as well as the interval from HAPLO-SCT1 to relapse significantly influenced both OS and LFS, confirming data obtained after other treatments for AL relapse post-transplant [27–29, 32–34]. No significant influence could be detected for adverse ELN risk classification, conditioning intensity and donor change for HAPLO-SCT2. Although not significant for OS/LFS, a MAC for HAPLO-SCT2 rendered a trend towards significance for lower RI and improved LFS, while it was not associated with a higher NRM. One could argue that a MAC for HAPLO-SCT2 may confer a survival advantage in carefully selected patients that are expected to tolerate higher intensity conditioning regimes. For this purpose, the transplant conditioning intensity (TCI) score might be helpful to choose a suitable conditioning regimen for HAPLO-SCT2, since this tool showed an improved categorization of different regimens concerning both intensity and toxicity, and is regarded as a valid improvement of the RIC/MAC stratification system [43]. Unfortunately, the limited number of patients did not allow for a reliable multivariate analysis of risk factors to further validate the role of MAC conditioning.

The cell source for HAPLO-SCT2 was BM in 18% of patients (36% at HAPLO-SCT1), which reflects the current trend towards PB as a stem cell source also for HAPLO-SCT in clinical practice due to easier harvesting, donor safety and comfort. Ruggeri et al. [44] had observed higher rates of acute GvHD in PB vs BM for HAPLO-SCT1 in AML/ALL, however, no difference in OS, LFS, NRM and GRFS were observed in this study. In contrast, Nagler et al. [45] showed higher GvHD and NRM rates, as well as inferior LFS and OS for HAPLO-SCT1 using PB compared to BM as the stem cell source in patients with ALL. In our study, there were no differences in 2-year NRM among recipients of BM and PB grafts after HAPLO-SCT2.

With respect to mechanisms behind post-transplant relapse after allo-SCT, loss of the mismatched HLA haplotype has been identified in about 1/3 of patients relapsing after HAPLO-SCT [40]. Obviously, HAPLO-SCT2 from the same donor as at HAPLO-SCT1 can be expected to be less effective after HLA loss. Since this phenomenon seems to occur more frequently among patients relapsing later than six months from HAPLO-SCT1 [36], we hypothesised that donor change might be advantageous among patients with late relapse, although data on HLA loss were not available in the patients analysed in this study. However, we were not able to detect an improved outcome after donor change among the 49 patients with a post-transplant remission of >6 months, which might be due to the low numbers as well as the fact that selection of a second HAPLO donor based on the investigation of HLA loss has only been introduced into clinical practice very recently.

Our study bears several limitations. First, the number of patients is limited despite covering a relatively long time period and having included all consecutive patients fulfilling the inclusion criteria. Besides the aspects discussed above, the low numbers might reflect a selection bias in our study population, including the risk not being representative of the entire population of patients relapsing after HAPLO-SCT. However, in general the patients investigated here represented a high-risk population with >1/3 of patients having suffered from early relapse, and 60% of patients having undergone HAPLO-SCT2 with an active disease. Hence, the cohort analysed here showed a disease-associated risk which was at least comparable to earlier studies on second SCT in other settings [27, 29, 32–34]. Nevertheless, the observed survival rates (2-year OS: 34%; 2-year LFS: 25%) are not inferior to those observed in these previous reports. Furthermore, we sought to report on patients and treatment characteristics with as many details as possible in order to allow for an exact comparison with published series as well as individual patients for whom HAPLO-SCT2 might be considered. Second, since PTCy was used for GvHD prophylaxis in 82% of HAPLO-SCT2, we were not able to separately study the influence of alternative strategies of GvHD prevention after HAPLO-SCT2, which, however, have been less frequently used across EBMT centres during recent years. Third, information on salvage treatment for relapse after HAPLO-SCT1 was not sufficient to include this aspect into the analysis. Recently, Piemontese et al. [46] described the overall treatment strategies applied for relapse after HAPLO-SCT1, but comparative analyses of the different salvage regimen were not possible even in this broader cohort. A thorough analysis of the short-term effects of the salvage treatment (remission, toxicity) and its influence in the outcome after HAPLO-SCT2 remains to be performed. Finally, as mentioned above, the limited number of patients did not allow for a reliable multivariate analysis of risk factors. Major risk factors already well established in other studies in different treatment settings were identified in our univariate analysis, suggesting that there are no substantial differences in the double HAPLO-SCT situation. Nevertheless, we might have missed less prominent factors influencing outcome, as well as the mutual dependence of the identified factors that might have been eliminated by multivariate testing.

Summarizing, within the limits of a retrospective registry-based analysis, our data show the feasibility of HAPLO-SCT2 after relapse post HAPLO-SCT1 with high engraftment rates and surprisingly low NRM rates. Outcome data as well as risk factors are comparable to results reported after allo-SCT2 in a matched donor setting. HAPLO-SCT2 is a viable option for AL patients relapsing after a HAPLO-SCT1. Nevertheless, there is still room for improvement. As discussed above, selection of a second HAPLO donor based on the presence or absence of HLA loss, as well as more efficient and less toxic strategies to achieve better disease control before HAPLO-SCT2, are among the options already available. Post-transplant maintenance strategies including novel drugs or additional cellular therapies should also be investigated after second transplantation including double HAPLO-SCT.

## Data Availability

The authors declare that all data generated or analysed during this study will be available to any researcher wishing to use them for non-commercial purposes on reasonable request, without breaching participant confidentiality.
